# The high-quality genome of lotus reveals tandem duplicate genes involved in stress response and secondary metabolites biosynthesis

**DOI:** 10.1093/hr/uhad040

**Published:** 2023-02-28

**Authors:** Huanhuan Qi, Feng Yu, Jiao Deng, Liangsheng Zhang, Pingfang Yang

**Affiliations:** State Key Laboratory of Biocatalysis and Enzyme Engineering, School of Life Sciences, Hubei University, Wuhan 430026, China; State Key Laboratory of Biocatalysis and Enzyme Engineering, School of Life Sciences, Hubei University, Wuhan 430026, China; State Key Laboratory of Biocatalysis and Enzyme Engineering, School of Life Sciences, Hubei University, Wuhan 430026, China; Research Center of Buckwheat Industry Technology, Guizhou Normal University, Guiyang 550001, China; College of Agriculture & Biotechnology, Zhejiang University, Hangzhou 310058, China; State Key Laboratory of Biocatalysis and Enzyme Engineering, School of Life Sciences, Hubei University, Wuhan 430026, China

Dear Editor,

Lotus (*Nelumbo nucifera* Gaertn.), which has been domesticated and cultivated for several thousands of years and endowed with religious and cultural symbolism [1], belongs to the *Nelumbo* genus Nelumbonaceae family. As an early eudicot, it is not only essential for plant phylogeny but also widely used as a vegetable, a medicinal herb, and for ornamental use. It contains abundant functional compounds, such as flavonoids and benzylisoquinoline alkaloids (BIAs), which are used to treat diverse diseases. A high-quality genome assembly is necessary to facilitate its breeding and efficient usage. Until now, two versions of the ‘China Antique’ (CA, wild lotus germplasm) genome have been released, which were assembled mainly through Illumina short-reads and annotated by transcriptome of short-reads data (CA v1, CA v2) [2, 3]. Recently, the genome of a cultivar ‘Taikonglian No.3’ (TK) has also been assembled [4]. Here based on 163.29 Gb PacBio long reads (N50 = 31.66 kb) data, the genome of CA was re-assembled using FALCON (v1.8.1) (https://github.com/PacificBiosciences/FALCON), with a total size of 817.9 Mb and contig N50 of 44.31 Mb (Fig. 1A). Additionally, a total of 90.23 Gb of clean Hi-C data was used to anchor the genome sequence through ALLHiC (https://github.com/tangerzhang/ALLHiC), and 807.37 Mb (98.7%) was anchored onto eight chromosomes (Table S1, see online supplementary material). The whole genome consisted of 70 scaffolds with N50 being 110.63 Mb (Fig. 1A)，of which eight were assembled as chromosomes, while the other 62 scaffolds could not be unambiguously assembled onto any of the eight chromosomes. Hereafter, it is named CA v3. About 99.29% of the Illumina reads were successfully aligned to CA v3, with a coverage of 99.83%. Benchmarking Universal Single-Copy Orthologs pipelines (BUSCO) demonstrated that 98.82% of the 1614 expected embryophytic genes could be aligned to the CA v3 genome. Furthermore, the Long Terminal Repeat (LTR) Assembly Index (LAI) value [5] and mapping rate of transcriptome data in 7 tissues were higher in CA v3 (Fig. 1A; Table S2, see online supplementary material). Compared to CA v2 and TK genomes, CA v3 had the best continuity and completeness (Fig. 1A).

**Figure 1:**
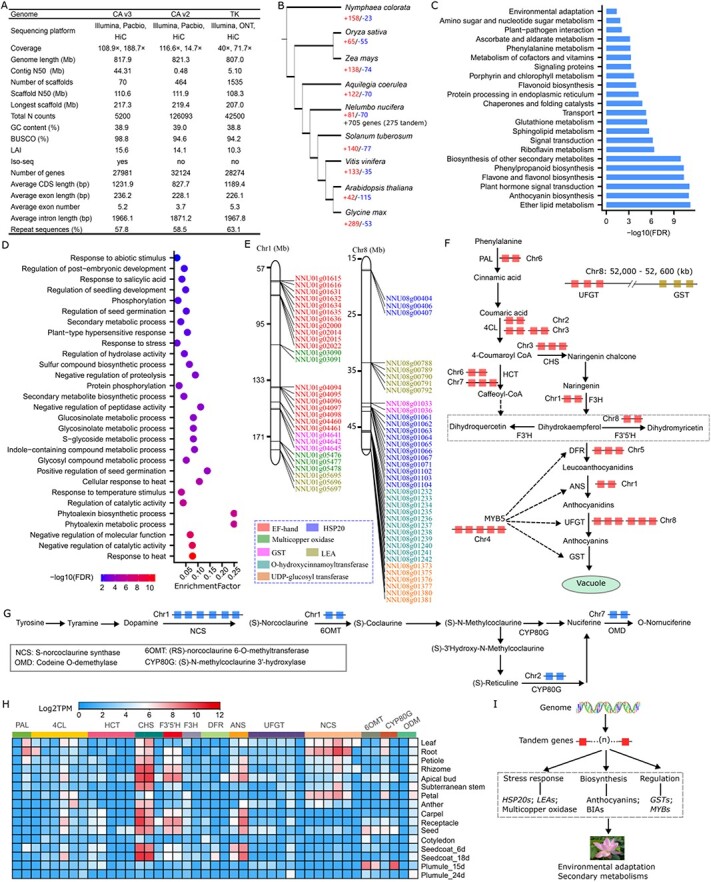
The genome of *N. nucifera*, arrangement, expression, and function of the expanded ortholog genes. **A** Comparison of CA v3 with CA v2 and TK genome assemblies. Iso-seq represented the long-read sequence of the transcriptome, and yes and no indicated whether Iso-seq data was used to predict protein-coding genes. CA v3, our current genome of CA; CA v2, the previously published genome of CA [3]; TK, the recently assembled genome of lotus cultivar ‘Taikonglian No.3’ [4]. BUSCO, Benchmarking Universal Single-Copy Orthologs. TK, ‘Taikonglian No.3’. LAI, the long terminal repeat assembly index. CDS, coding sequence. **B** The number of expanded orthogroups and genes in lotus. OrthoFinder (v2.5.2) was applied to analyse orthologous, and Cafe (v5.0) was used to calculate the significance of the identified orthogroups. The red digit represents the number of expanded orthogroups, the blue digit represents the number of contracted orthogroups, and the black digit below represents the number of expanded genes. **C** KEGG enrichment analysis of expanded genes in lotus. FDR, adjusted *P*-value. **D** GO enrichment analysis of 275 tandem duplicate genes of expanded genes. **E** Physical distribution of tandem duplicate genes located in chromosome 1 and chromosome 8. LEA, late embryogenesis abundant protein; GST, glutathione S-transferase. **F** The tandem duplicate genes involved in anthocyanin biosynthesis. 4CL, 4-coumarate—CoA ligase; ANS, anthocyanidin synthase; CHS, chalcone synthase; DFR, dihydroflavonol 4-reductase; F3H, flavanone 3-hydroxylase; F3′5′H, flavonoid 3′,5′-hydroxylase; HCT, shikimate O-hydroxycinnamoyl transferase; PAL, phenylalanine ammonia-lyase; UFGT, anthocyanidin 3-O-glucosyltransferase. **G** The tandem duplicate genes are involved in alkaloid biosynthesis. **H** The expressional patterns of tandem duplicate genes involved in the biosynthesis of anthocyanin and benzylisoquinoline alkaloids. **I** The putative model of tandem duplicate genes involved in environmental adaptation and secondary metabolites biosynthesis.

Based on the Extensive *de novo* TE annotator pipeline, a total of 472.36 Mb (57.75%) repetitive sequences in CA v3 were identified, with Copia and Gypsy of LTR elements accounting for 18.27% and 11.09%, respectively (Fig. S1, see online supplementary material). Through a combination of *de novo*-, homolog-, and transcriptome-based strategies (seven tissues of RNA-Seq data and 30 Gb of Iso-Seq data), 27 981 high-confident genes were predicted (Fig. 1A), of which 99.3%were successfully annotated in NR, Swiss-Prot, Kyoto encyclopedia of genes and genomes (KEGG), and InterPro databases. Over 96% and 50% of the predicted genes could be detected at the mRNA and protein levels, respectively (Fig. S1, see online supplementary material). The average lengths of coding sequences and exons in CA v3 were longer than those in CA v2 and TK (Fig. 1A). The lengths of gene and protein in CA v3 were longer than those in CA v2, and the lengths of exon and intron were also longer in CA v3 (Fig. S2, see online supplementary material). These data collectively demonstrated that our newly assembled genome had higher quality and could be used for further comparative genome analysis.

For comparative genome analysis, eight genomes from early angiosperm, monocot, early eudicot, and eudicot plants were collected to identify the expanded genes in the lotus genome through OrthoFinder (v2.5.2) and Cafe (v5.0) (Fig. 1B). The phylogenetic relationship showed that *N. nucifera* diverged after monocots, and before eudicots differentiation. A total of 81 orthogroups were significantly expanded in *N. nucifera*, which contained 705 protein-coding genes (Fig. 1B). KEGG analysis of expanded genes showed that those involved in phenylpropanoid and flavonoids (such as anthocyanin, flavone, and flavonol) biosynthesis, glutathione metabolism, chaperones, and protein folding, and environmental adaptation were significantly enriched (Fig. 1C). These genes mainly encode EF-hand, multicopper oxidase, heat shock protein 20 (HSP20), glutathione S-transferase (GST), late embryogenesis abundant protein (LEA), O-hydrocinnamoyltranse (HCT), and UDP-glucoronosyl transferase (UGT), and are highly related to the typical biological features of *N. nucifera*. Interestingly, most of them were distributed as gene clusters within adjacent genomic regions.

The colinear homologs and duplicated regions of the CA v3 genome were calculated through MCScanX (https://github.com/wyp1125/MCScanX) [6], which detected 2456 tandem duplicate genes in the whole genome, whereas a much higher ratio (275 among the 705, *chi*-test, *P* < 2.2 × 10^−16^) of the expanded genes were tandem duplicate genes. Gene ontology (GO) analysis of 275 genes showed that these were mainly involved in stress response and secondary metabolite biosynthesis process (Fig. 1D). Of expanded genes that were involved in enriched pathways, more than 75% of them in chr1 and chr8 were tandem duplicate genes (Fig. 1E). The enriched functional groups of all tandem duplicate genes were similar to those obtained from the expanded genes (Fig. S3, see online supplementary material), indicating that tandem duplicate genes contribute a lot to lotus biological features. Specifically, tandem duplicate genes in the anthocyanin biosynthetic pathway, including those encoding the enzymes catalyzing the reactions from phenylalanine to anthocyanin [7] were investigated (Fig. 1F; Table S3, see online supplementary material). A total of nine enzymes were encoded by tandem duplicate genes, in which there were six genes for both 4-coumarate—CoA ligase and anthocyanidin 3-O-glucosyltransferase (UFGT), five for O-hydroxycinnamoyltransferase, three for chalcone synthase (CHS) and dihydroflavonol 4-reductase (DFR), and two for phenylalanine ammonia-lyase (PAL), flavanone 3-hydroxylase (F3H), anthocyanidin synthase (ANS) and flavonoid 3′,5′-hydroxylase (F3’5’H), respectively. Furthermore, 19 genes encoded glutathione S-transferase (GST) responding to anthocyanin transportation into the vacuole were also detected as tandem duplicate genes (Table S4, see online supplementary material). The transcription factor MYB5 (NNU04g01662) that regulated anthocyanin biosynthesis [8] was located within a tandem region comprising five *MYB* genes, and another 23 *MYB* genes were also tandemly repeated (Fig. 1F; Table S4, see online supplementary material). Although six tandem duplicate genes of *UFGT* in anthocyanin biosynthesis were detected, an additional 24 UGT encoding genes were also tandemly repeated (Tables S3 and S4, see online supplementary material). Furthermore, different genes in this pathway were found located within the 600 kb region in chr8 (Fig. 1F). Along with the higher expression correlation between genes involved in anthocyanin biosynthesis and genes encoding GSTs, MYBs, and UGTs (Fig. S4, see online supplementary material), it seems that tandem duplicate genes commonly occurred in biosynthesis, transport, and regulation of anthocyanin in lotus.

Alkaloids, such as nuciferine, which is synthesized from BIAs, are another type of typical compound in lotus [9]. The distribution of genes encoding the enzymes in the pathway was also analysed and found that genes encoding S-norcoclaurine synthase (NCS), (RS)-norcoclaurine 6-O-methyltransferase (6OMT), (S)-N-methylcoclaurine3’-hydroxylase (CYP80G), and codeine O-demethylase (OMD) were mostly tandem duplicate genes on chr1, chr2, and chr7, respectively (Fig. 1G; Table S3, see online supplementary material). These collectively indicated that tandem duplication was a vital component in stress-responsive genes and secondary metabolic genes. Tandem duplication of these genes might be able to ascertain the occurrence of the biological events they are involved in. Moreover, similar expression patterns of most clustered genes, such as *NCSs*, *CHSs*, *F3’5’Hs*, *LEAs,* and *HSP20s* were detected in diverse developmental tissues (Fig. 1H; Fig. S5, see online supplementary material), implying the potential co-expression of clustered genes. Co-localization and co-expression of clustered homologous genes in metabolic pathways of lotus features ensure well-organized biosynthesis and regulation.

In summary, our *de novo* assembly of *N. nucifera* genome with higher quality provides robust references to clarify gene distribution and identify genes controlling environmental adaptation and secondary metabolites biosynthesis (Fig. 1I). It is also a new resource for functional genomic study and genetic improvement in lotus. To make the genomic and related data publicly available, we constructed a website for our newly assembled genome CA v3 (lotus-db.cn) based on the Drupal platform, in which users can browse the genome through JBrowse, search the homologous protein, retrieve sequence, run BLAST program, and acquire gene expression data at mRNA and protein levels. This will promote and accelerate its application.

## Acknowledgments

We would like to acknowledge Rebecca Njeri Damaris for revising the manuscript. This work was supported by the National Natural Science Foundation of China (NSFC no. 32102422).

## Author contributions

P.Y. and L.Z. conceived and designed the project. H.Q. and F.Y. performed data analysis. H.Q. drafted the manuscript. J.D., L.Z., P.Y., and F.Y. discussed and revised the manuscript. All authors contributed to and approved the final manuscript.

## Data availability

The whole genome sequence data reported in this paper have been deposited in the Genome Warehouse in the National Genomics Data Center [10], Beijing Institute of Genomics, Chinese Academy of Sciences / China National Center for Bioinformation, under accession number GWHBQCP00000000 that is publicly accessible at https://ngdc.cncb.ac.cn/gwh.

## Conflict of interest

The authors declare there is no conflict of interest.

## Supplementary data

Supplementary data is available at *Horticulture Research* online.

## Supplementary Material

Web_Material_uhad040
