# Supplementary figures and images for: The high-quality genome of lotus reveals tandem duplicate genes involved in stress response and secondary metabolites biosynthesis
Source: Hortic Res. 2023 Feb 28;10(5):uhad040. doi: 10.1093/hr/uhad040 (PMC10163359; doi:10.1093/hr/uhad040)

Figure S1

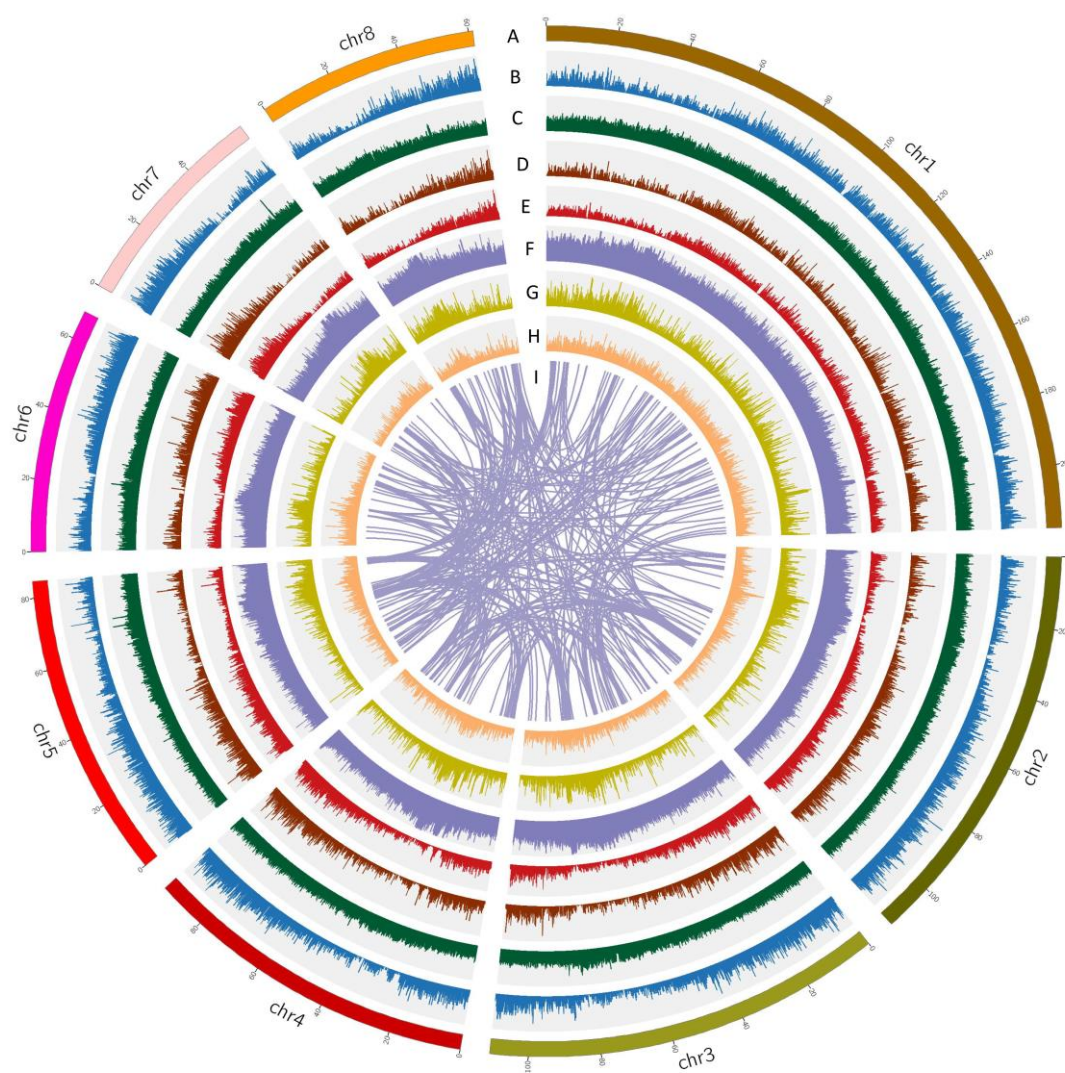

Figure S2

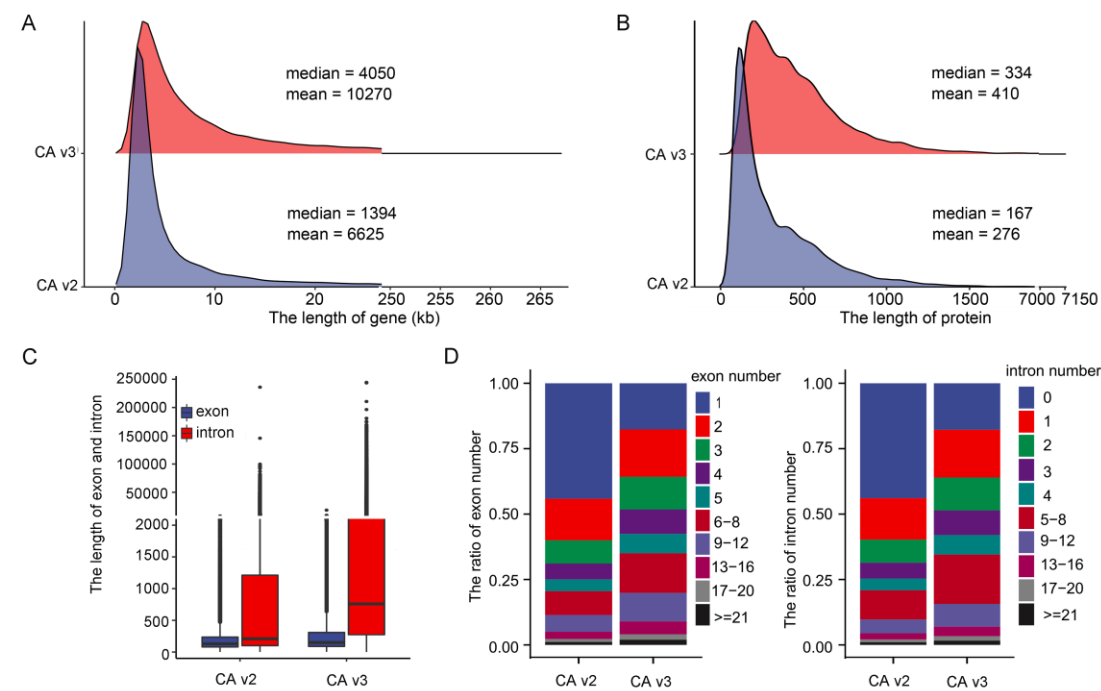

**Figure S3**

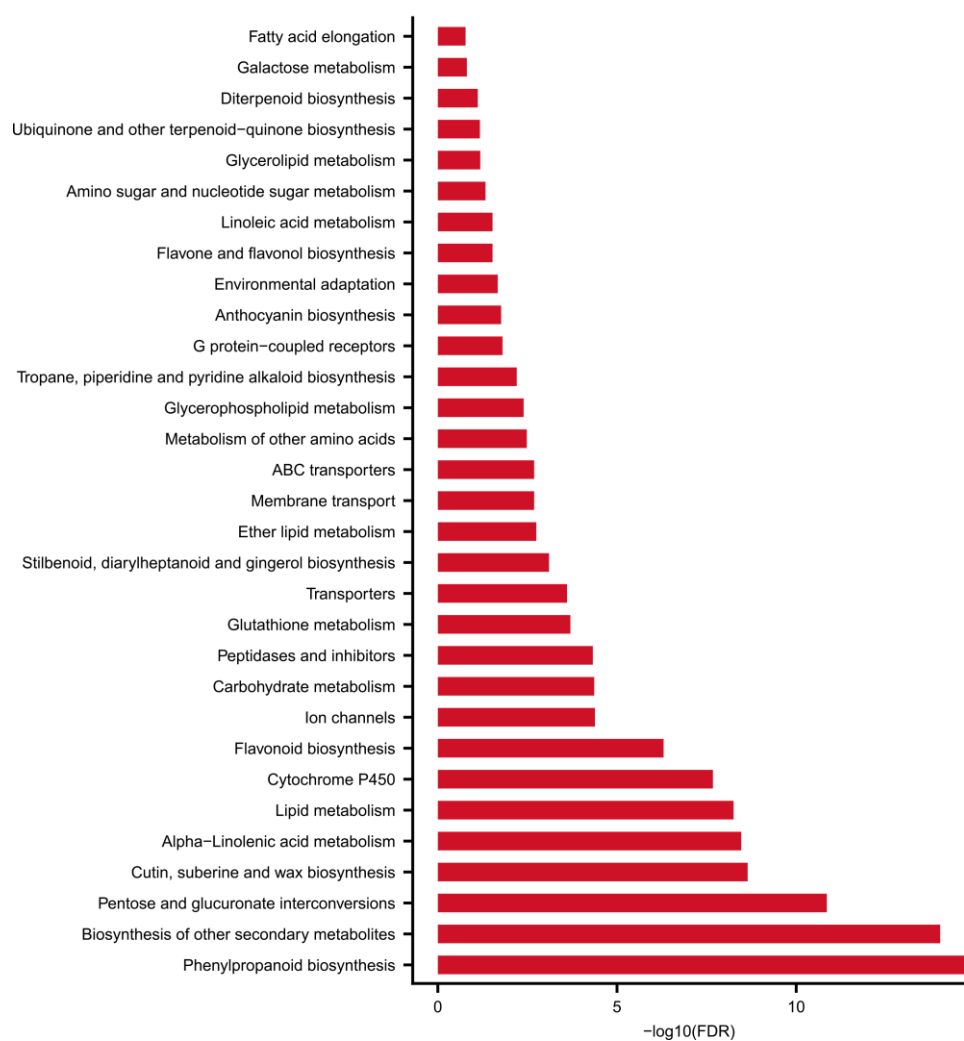

**Figure S4**

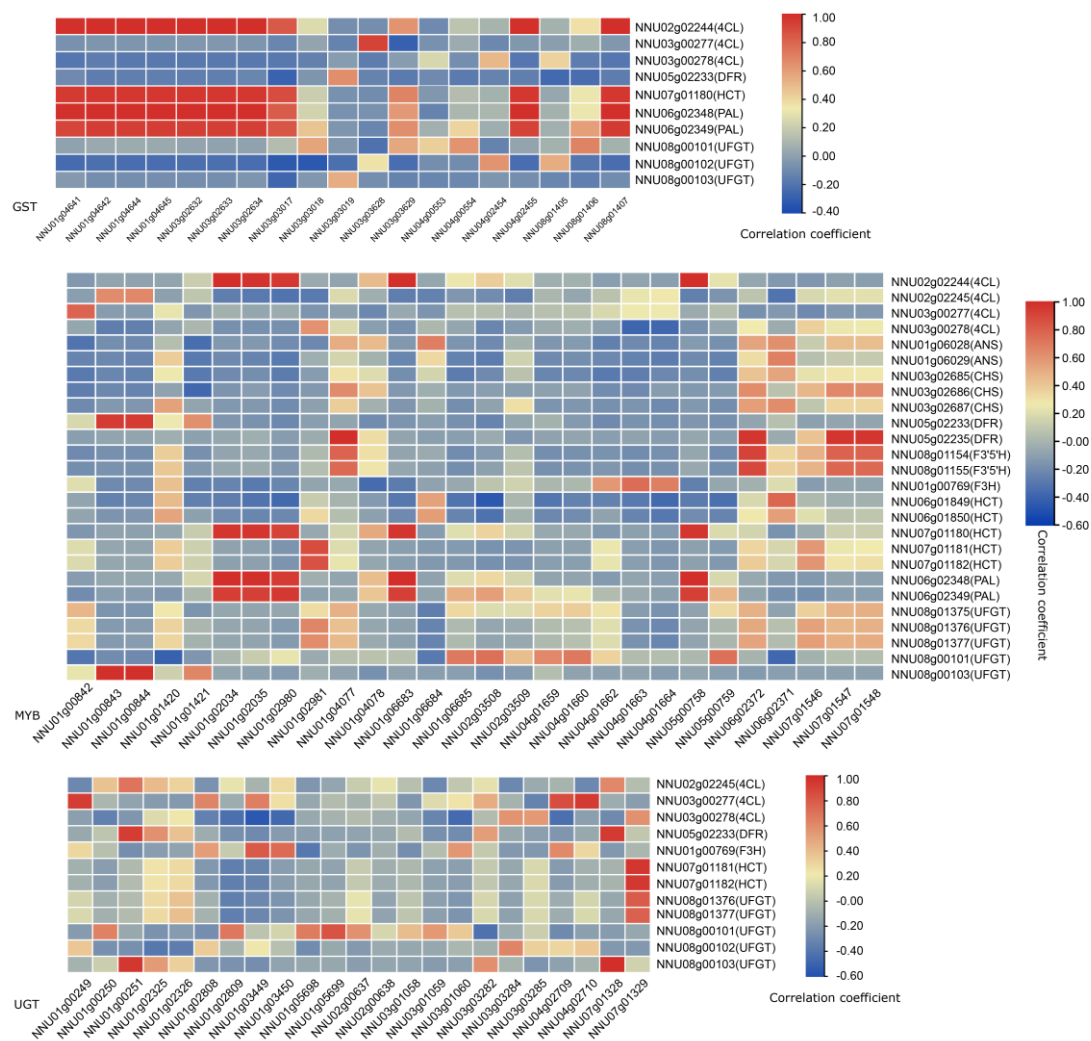

Figure S5

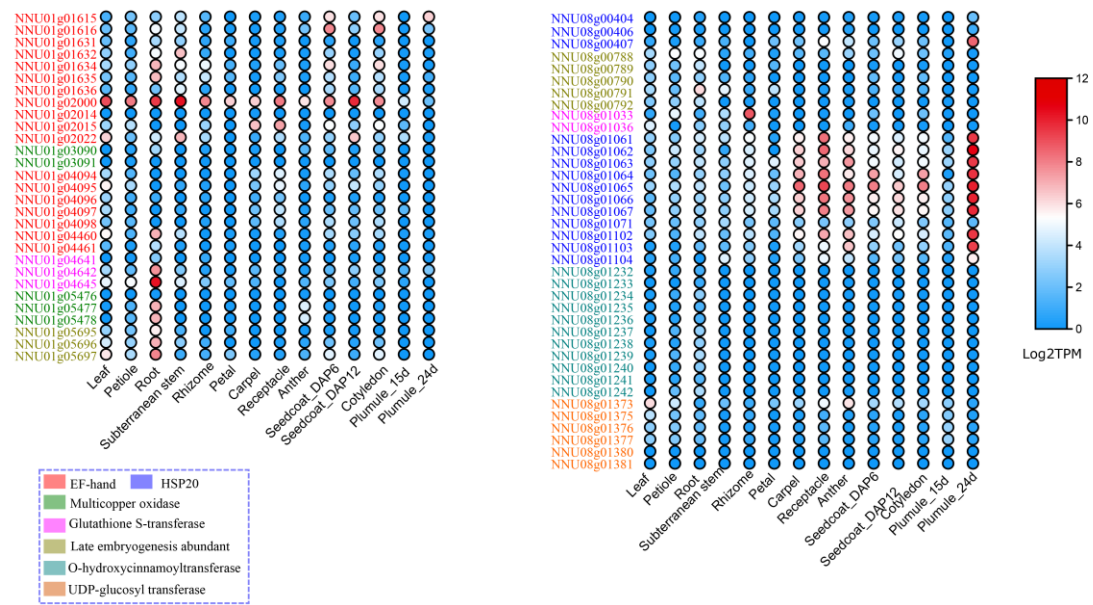

Supplement: Web_Material_uhad040 [file web_material_uhad040.zip › Figure S1-S5.pdf]
